# King's Fund Emergency Distribution

**Published:** 1920-07-10

**Authors:** 


### £250,000 FOR LONDON HOSPITALS.

### King's Fund Emergency Distribution.

The following are the details of the emergency distribution of £250,000 made by King Edward's Hospital Fund to assist the London Hospitals, having regard to their pressing needs.

The grants are made with the object of assisting the hospitals to carry on their work; and it is the hope of the Fund that, where the Committee of Management are experiencing, or have reason to anticipate, any special difficulty in raising funds for maintenance, the grant may give them both the opportunity and the encouragement to consider methods of improving the finances, and in particular (*a*) to consider whether the income of the hospital can, without detriment, be increased by taking some special steps—*e.g.*, by the organisation of contributions from employers or from employed, or from patients who can afford to pay, or by other methods; (*b*) to take whatever steps may be necessary and practicable to keep the expenditure within the available or prospective income—*e.g.*, by postponing bed extensions, and by exercising more stringent control over cost of working, especially where the cost is substantially in excess of the average of the hospitals in the same group in the Fund's statistical report.

The Committees of Management are asked to communicate to the Fund in due course particulars of any action which they may decide to take.

List of Grants.

Acton Hospital, £100; Alexandra Hospital for Children, £100. Beckenham Cottage Hospital, £25; Belgrave Hospital for Children, £600; Blackheath and Charlton Hospital, £100; Bolingbroke Hospital, £1,500; British Hospital for Mothers and Babies, Woolwich, £120. Canning Town Women's Settlement Hospital (late Medical Mission), £500; Central London Ophthalmic Hospital, £300; Central London Throat and Ear Hospital, £100; Charing Cross Hospital, £2,100; Chelsea Hospital for Women, £500; Cheyne Hospital for Sick and Incurable Children, £200; City of London Hospital for Diseases of the Chest (Victoria Park), £3,000; City of London Maternity Hospital, £700; Clapham Maternity Hospital, £450. Dreadnought Hospital, £1,000. East End Mothers' Lying-in Home, £450; East Ham Hospital, £150; East London Hospital for Children, £3,000; Elizabeth Garrett Anderson Hospital, £1,250; Eltham and Mottingham Cottage Hospital, £50. Finchley Cottage Hospital, £50; Florence Nightingale Hospital for Gentlewomen, £300; French Hospital, £100. General Lying-in Hospital, £500; Great Northern Central Hospital, £12,000; Grosvenor Hospital for Women, £400; Guy's Hospital, £7,000. Hampstead General and North-West London Hospital, £2,000; Hendon Cottage Hospital, £25; Hornsey Cottage Hospital, £25; Hospital for Consumption, Brompton (including sanatorium at Frimley), £3,000; Hospital for Diseases of the Throat, £50; Hospital for Epilepsy and Paralysis, £1,200; Hospital for Sick Children, £8,000; Hospital for Women (Soho Square), £2,000; Hospital of St. John and St. Elizabeth, £100. Infants' Hospital, £700; Italian Hospital, £400. Kensington and Fulham General Hospital, £25; Kensington Dispensary and Children's Hospital, £25; King Edward Memorial Hospital (Ealing), £750; King's College Hospital, £17,500. London Fever Hospital, £500; London Homœopathic Hospital, £3,500; London Hospital, £35,000; London Lock Hospital, £1,000; London Temperance Hospital, £3,500. Maternity Charity and District Nurses' Home, Plaistow, £250; Memorial Hospital, Mildmay Park, £200; Metropolitan Hospital, £11,000; Middlesex Hospital, £10,000; Middlesex Hospital Cancer Charity, £500; Mildmay Mission Hospital, £600; Miller General Hospital for South-East London, £4,000; Mothers' Hospital of the Salvation Army, £250. National Hospital for Diseases of the Heart, £200; National Hospital for the Paralysed and Epileptic, £8,000; Nelson Hospital (late South Wimbledon, etc.), £300; Northcourt Hospital and Home for Sick Children, £100; Norwood Cottage Hospital, £225. Paddington Green Children's Hospital, £250; Passmore Edwards Hospital for Willesden, £1,000; Passmore Edwards Hospital for Wood Green, etc., £150; Poplar Hospital for Accidents, £250; Prince of Wales's General Hospital, £7,000. Queen Charlotte's Lying-in Hospital, £2,000; Queen Mary's Hospital for the East End, incorporating West Ham and Eastern General Hospital, £6,000; Queen's Hospital for Children, £6,500. Royal Chest Hospital (City Road), £1,000; Royal Dental Hospital of London, £500; Royal Eye Hospital, £500; Royal Free Hospital, £6,000; Royal Hospital, Richmond, £500; Royal London Opthalmic Hospital, £3,000; Royal National Orthopædic Hospital, £2,000; Royal Waterloo Hospital for Children and Women, £2,000; Royal Westminster Opthalmic Hospital, £200. St. Andrew's Hospital (Dollis Hill), £50; St. Columba's Hospital, £200; St. George's Hospital, £8,500; St. John's Hospital, Lewisham, £350; St. John's Hospital for Diseases of the Skin, £100; St. Luke's Hospital for Advanced Cases, £100; St. Mark's Hospital, £225; St. Mary's Hospital, £2,000; St. Mary's Hospital for Women and Children, Plaistow, £1,500; St. Monica's Home Hospital, £100; St. Peter's Hospital for Stone, £300; St. Saviour's Hospital for Ladies of Limited Means (Osnaburgh Street), £100; St. Thomas's Hospital, £12,000; Samaritan Free Hospital for Women, £1,500; Santa Claus Home, £30; South-Eastern Hospital for Children (Sydenham), £200; South London Hospital for Women, £500; Stoke Newington Home Hospital for Women, £75. University College Hospital, £10,000. Victoria Hospital for Children, £1,500. Walthamstow, Wanstead, and Leyton Children's and General Hospital, £350; West End Hospital for Nervous Diseases, £750; Western Ophthalmic Hospital, £100; West London Hospital, £9,500; Westminster Hospital, £9,500; Wimbledon Hospital, £50. Total, £250,000.

